# Differentially methylated regions identified in bovine embryos are not observed in adulthood

**DOI:** 10.1590/1984-3143-AR2022-0076

**Published:** 2023-03-13

**Authors:** Luna Nascimento Vargas, Allice Rodrigues Ferreira Nochi, Paloma Soares de Castro, Andrielle Thainar Mendes Cunha, Thainara Christie Ferreira Silva, Roberto Coiti Togawa, Márcia Marques Silveira, Alexandre Rodrigues Caetano, Maurício Machaim Franco

**Affiliations:** 1 Laboratório de Reprodução Animal, Embrapa Recursos Genéticos e Biotecnologia, Brasília, DF, Brasil; 2 Instituto de Biotecnologia, Universidade Federal de Uberlândia, Uberlândia, MG, Brasil; 3 Agência de Defesa Agropecuária do Paraná, Curitiba, PR, Brasil; 4 Embrapa Recursos Genéticos e Biotecnologia, Brasília, DF, Brasil; 5 Faculdade de Medicina Veterinária, Universidade Federal de Uberlândia, Uberlândia, MG, Brasil

**Keywords:** epigenetics, reprogramming, methylome, DMRs, cattle

## Abstract

The establishment of epigenetic marks during the reprogramming window is susceptible to environmental influences, and stimuli during this critical stage can cause altered DNA methylation in offspring. In a previous study, we found that low levels of sulphur and cobalt (low S/Co) in the diet offered to oocyte donors altered the DNA methylome of bovine embryos. However, due to the extensive epigenetic reprogramming that occurs during embryogenesis, we hypothesized that the different methylation regions (DMRs) identified in the blastocysts may not maintain in adulthood. Here, we aimed to characterize DMRs previously identified in embryos, in the blood and sperm of adult progenies of two groups of heifers (low S/Co and control). We used six bulls and characterized the DNA methylation levels of *KDM2A*, *KDM5A*, *KMT2D*, and *DOT1L* genes. Our results showed that all DMRs analysed in both groups and tissues were hypermethylated unlike that noticed in the embryonic methylome profiles. These results suggest that embryo DMRs were reprogrammed during the final stages of *de novo* methylation during embryogenesis or later in development. Therefore, due to the highly dynamic epigenetic state during early embryonic development, we suggest that is essential to validate the DMRs found in embryos in adult individuals.

## Introduction

DNA methylation has been studied in embryos of various species ever since techniques were first developed for the analysis of DNA methylation (Stevens et al., 1988; Kafri et al., 1992; Kang et al., 2001). The technological advancements of the past few decades have made it possible to access the embryonic methylome through whole-genome sequencing. Several studies that have analysed DNA methylation during embryogenesis in cattle, sheep, and humans have since been published (Guo et al., 2014; Zhu et al., 2018; Duan et al., 2019; Zhou et al., 2019; Zhang et al., 2021b). Although these analyses of the various embryonic stages have provided valuable information to elucidate some regulatory mechanisms, the epigenetic state during the early embryonic development is highly dynamic and requires further study.

During the initial stages of development in mammals, particularly gametogenesis and embryogenesis, extensive epigenetic reprogramming occurs to support proper embryonic and foetus growth (Reik et al., 2001; Canovas et al., 2017). First, a wide loss of DNA methylation is initiated during primordial germ cell (PGC) formation in the foetal phase (Reik et al., 2001). After demethylation, a subsequent *de novo* DNA methylation process occurs, establishing a new sex-specific pattern in developing gametes (Lee et al., 2002). This *de novo* methylation process differs between male and female germ lines. In the male germ line, the process is initiated in the foetus; hence, the paternal allele is hypermethylated at birth in this cell lineage (Davis et al., 2000). In the female germ line by contrast, the process is arrested during meiosis in the foetal period, and *de novo* methylation begins only after birth during folliculogenesis/oogenesis, which is around puberty (Obata et al., 1998; Fagundes et al., 2011). A follicle is recruited for growth, and *de novo* DNA methylation is initiated in the oocytes. However, the process is not completed without the aid of appropriate hormonal stimuli.

During embryogenesis, the parental pronucleus undergoes a differential demethylation process, where the paternal genome is significantly demethylated by an active mechanism closely following fertilization (Oswald et al., 2000). The maternal genome, however, loses DNA methylation at a later stage due to cleavage divisions through a passive mechanism (Sasaki and Matsui, 2008). After DNA demethylation, global *de novo* methylation begins at the 8-16 cell stage in cattle (Dean et al., 2001; Ivanova et al., 2020). At the blastocyst stage, where several methylome analyses takes place, *de novo* methylation has been started; however, a wide range of reprogramming continues until the establishment of DNA methylation patterns in the embryonic and extra-embryonic tissue (Greenberg and Bourc'his, 2019). Therefore, several mechanisms of epigenetic remodeling still happen from the blastocyst stage until the establishment of the tissue epigenome of the adult animal (Xu et al., 2021). Now, how informative could the methylome of the embryos be if the DNA methylation patterns are analysed before the structures have been completely reprogrammed?

One of the main reasons for the increased interest in embryonic DNA methylation is the Developmental Origins of Health and Disease (DOHaD) study and the long-term consequences for the progeny, which is a crucial concern for humans (Almeida et al., 2019; Lapehn and Paquette, 2022). The DOHaD theory states that the foetus undergoes an intrauterine environmental adaptation process in order to cope with those same conditions following birth (Wadhwa et al., 2009; Moreno-Fernandez et al., 2020). Therefore, adverse environmental stimuli during foetal programming can affect the establishment of epigenetic marks. As the offspring may not face the same conditions after birth, these adaptions can lead to susceptibility to diseases in adulthood (Cropley et al., 2006; Martinez et al., 2014).

As a result of several studies in humans and animal models, the maternal diet during early pregnancy is known to affect the embryo and long-term conceptus (Tobi et al., 2015; Wang et al., 2015; Finer et al., 2016; Knight et al., 2018; Serrano-Perez et al., 2020). We found that low levels of sulphur and cobalt in the diet offered to oocyte donors altered the DNA methylome of bovine embryos (Nochi et al., 2022). The inheritance of differential methylation regions (DMRs) by the next generation is known as an intergenerational epigenetic inheritance (Skvortsova et al., 2018). Analysing embryonic methylomes can be helpful for the analysis of epigenetic inheritance as an initial screening strategy. However, caution must be exercised when considering the information extracted from these data and when projecting the embryonic methylome onto adult tissues (Nochi et al., 2022).

Thus, this study tests the hypothesis that the DMRs identified in the embryos are not maintained in the somatic tissues of the animal in adulthood, considering that the embryos have a high probability of losing these DMR patterns during the second wave of epigenetic reprogramming, which occurs during early development (Reik et al., 2001). Accordingly, we aimed to characterize four DMRs in genes, which were previously identified in embryos and are involved in the epigenetic machinery, in the blood and sperm of adult progenies of two groups of heifers used in a related previous study in our laboratory (Nochi et al., 2022). These genes of special interest are related to histone methylation: writer lysine methyltransferase 2D (*KMT2D),* DOT1-like histone lysine methyltransferase (*DOT1L)*, erasers lysine demethylase 2A (*KDM2A),* and lysine demethylase 5A (*KDM5A)*.

## Methods

### Ethical approval

This experimental study has been approved by the Ethics Committee on Animal Use (CEUA-Protocol n° 98/2010), School of Veterinary Medicine and Animal Science, Universidade Estadual Paulista “Júlio de Mesquita Filho.”

### Animals and experimental diets

In this study, we used animals from a previous study conducted in our laboratory (Nochi et al., 2022). Briefly, the heifers were separated into groups with different diets, the control and the group with low sulfur and cobalt (low S/Co). The respective diets were offered to the animals for six months (pre- and periconceptional periods). At the end of the experiment, the heifers were inseminated with the same bull used for the *in vitro* embryo production (IVP) performed in Nochi et al. (2022). Among the progeny of those heifers, we collected the blood and sperm of the bulls in adulthood.

### Sample collection

The blood and sperm cells were collected from six bulls (Bull 1, Bull 2, Bull 3, Bull 4, Bull 5, and Bull 6) — the progenies of heifers (two from the control and four from the low S/Co group). Semen from six Nellore bulls (*Bos taurus indicus*) was collected from the ejaculate via electroejaculation. Sperm quality, concentration, motility, plasma membrane integrity, and morphology were evaluated (Table 1). Semen samples were stored in liquid nitrogen (-196 ºC) until DNA isolation was performed.

**Table 1:** Concentration (×10^6^/mL), total motility (%), plasma membrane integrity (%), and sperm normal morphology (%) in the semen of each animal before freezing.

	**Animals**					
	**Bull 1**	**Bull 2**	**Bull 3**	**Bull 4**	**Bull 5**	**Bull 6**
**Concentration (**×**10^6^/mL)**	1710	1510	1250	2920	650	1750
**Total motility (%)**	20%	90%	70%	90%	90%	90%
**Plasma membrane integrity (%)**	43%	79%	82%	66,5%	78%	61%
**Sperm normal morphology (%)**	31%	80,5%	66,5%	67,5%	64,5%	67%

### DNA isolation

Genomic DNA was isolated from white blood cells using the DNeasy Blood & Tissue Kit (Qiagen, CA, USA) according to the manufacturer’s instructions. Sperm DNA was isolated using a protocol based on salting out as described by Carvalho et al. (2012). The DNA samples were stored at -20 °C for sodium bisulphite treatment. The quality of the DNA samples was evaluated using agarose gel electrophoresis.

### Sodium bisulphite treatment

Blood and sperm genomic DNA (500 ng) were treated with sodium bisulphite using the EZ DNA Methylation-Lightning kit (Zymo Research, Irvine, CA, USA), according to the manufacturer’s instructions. Sodium bisulphite-treated DNA were stored at -80 °C until PCR amplification was performed.

### Bisulphite PCR

**Figure 1:**
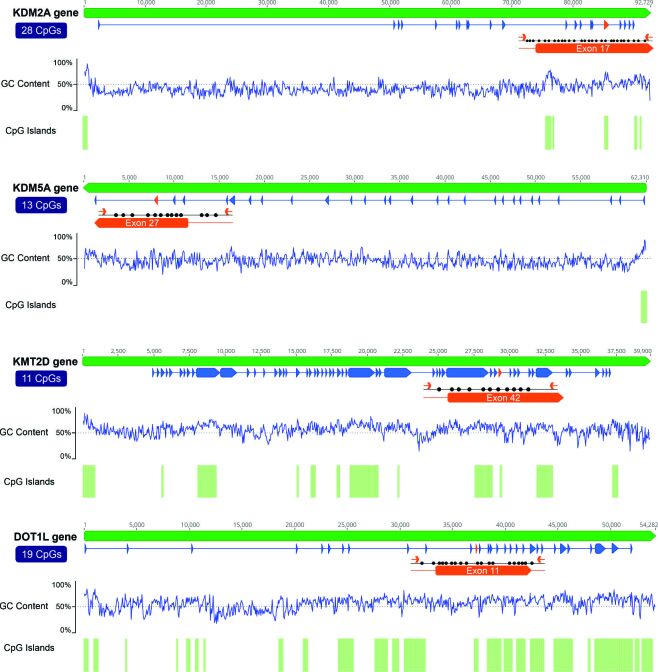
Representation of the *KDM2A*, *KDM5A*, *KMT2D,* and *DOT1L* gene structures, GC content, and CpG island prediction. Green bars represent the input sequence; below, blue lines represent introns, blue arrows represent exons, and orange arrows represent primer positions. The GC content and CpG islands are predicted for each gene. The graphs were generated using Geneious v2020.0.5 (Biomatters, Auckland, New Zealand).

PCR was performed to amplify the DMRs in the genes *KDM2A*, *KDM5A*, *KMT2D*, and *DOT1L,* which are associated with histone-active methylation marks (Nochi et al., 2022). Primers were designed using the MethPrimer (Li and Dahiya, 2002) and Bisulphite Primer Seeker software (Zymo Research) to flank the DMRs, which were located on CpG islands in all genes except KDM5A (Figure 1).

The primer sequences, GenBank accession numbers, number of CpG sites, amplicon sizes, and annealing temperatures are listed in Table 2. The total volume of each reaction prepared was 20 μL and comprised of 1× Taq buffer, 1.5 mM MgCl_2_, 0.4 mM dNTPs, 1 U Platinum™ Taq polymerase (Invitrogen, CA, USA), 0.5 μM primers (forward and reverse), and 2 μL of bisulphite-treated DNA. PCR was performed with an initial denaturing step at 94 °C for 3 min, followed by 29 cycles of 94 °C for 40 s, annealing (Table 2) for 1 min, and 72 °C for 1 min. The final extension was at 72 °C for 15 min. After PCR, amplicons were purified from agarose gels using the Wizard^®^ SV Gel and PCR Clean-Up System (Promega, Madison, WI, USA), according to the manufacturer’s instructions.

**Table 2:** Primers for methylation analysis.

**Gene**		**Primer Sequence (5’-3’)**	**Genbank acession number**	**CpG sites**	**Amplicon length (bp)**	**Annealing (°C)**
KDM2A	F:	GGTAAGTGTAGAGGGTTTTGAAGAAAGGAGATATTG	540141	28	387	60
	R:	TTAACTTTCTCAACTTCAAACAACTCCTTTTTACC				
KDM5A	F:	AAATTGGTTAAGAAGTTAGTAAAAGAAGAAGAGAG	507962	13	334	55
	R:	ATAATACAAAACCAAATCCTAAAATCAAAACAAACC				
KMT2D	F:	TAGTTAGAGTGGAGTAGATTTTGTGGGGTTT	506805	11	333	60
	R:	CACAACTAAAAACCAAACTACCCCCTTATC				
DOT1L	F:	GTTATGGGTATTTTTTAGGTTGGTGGTTG	510442	19	335	60
	R:	TACAAAATAAAAACCATATTCCAAACCCAC				

F (forward); R (reverse); bp (base pair).

### Cloning and bisulphite sequencing

The purified amplicons were cloned into the TOPO TA Cloning^®^ vector (Invitrogen, CA, USA) and transferred into DH5α cells using a heat shock procedure. Plasmid DNA was isolated using Pure Yield Plasmid Miniprep (Promega, Madison, WI, USA), and individual clones were sequenced using BigDye^®^ cycle sequencing chemistry and an ABI3100 automated sequencer (Applied Biosystems, Foster City, CA). Electropherogram quality was analysed using Chromas^®^ (Technelysium Pty Ltd, South Brisbane, Australia), and methylation patterns were processed using the QUantification tool for Methylation Analysis (QUMA) (Kumaki et al., 2008). DNA sequences were compared with GenBank reference sequences (Table 2), and only those sequences originating from clones with ≥ 95% identity and ≥ 97% cytosine conversion were used in the analysis (n = 684). The efficiency of the bisulphite treatment was calculated based on the percentage of CpH (H = A, C, or T) site conversion divided by the total number of CpH sites in the sequence.

### Statistics analysis

Comparison of methylation data between two groups was done using the Mann-Whitney test and more than two groups were performed using the Kruskal-Wallis test followed by Dunn’s multiple comparison test. Comparative methylation analysis of CpG site was performed using Fisher's exact test. Statistical significance was set at p < 0.05. Data analyses were performed using QUMA and GraphPad Prism software.

## Results

**Figure 2:**
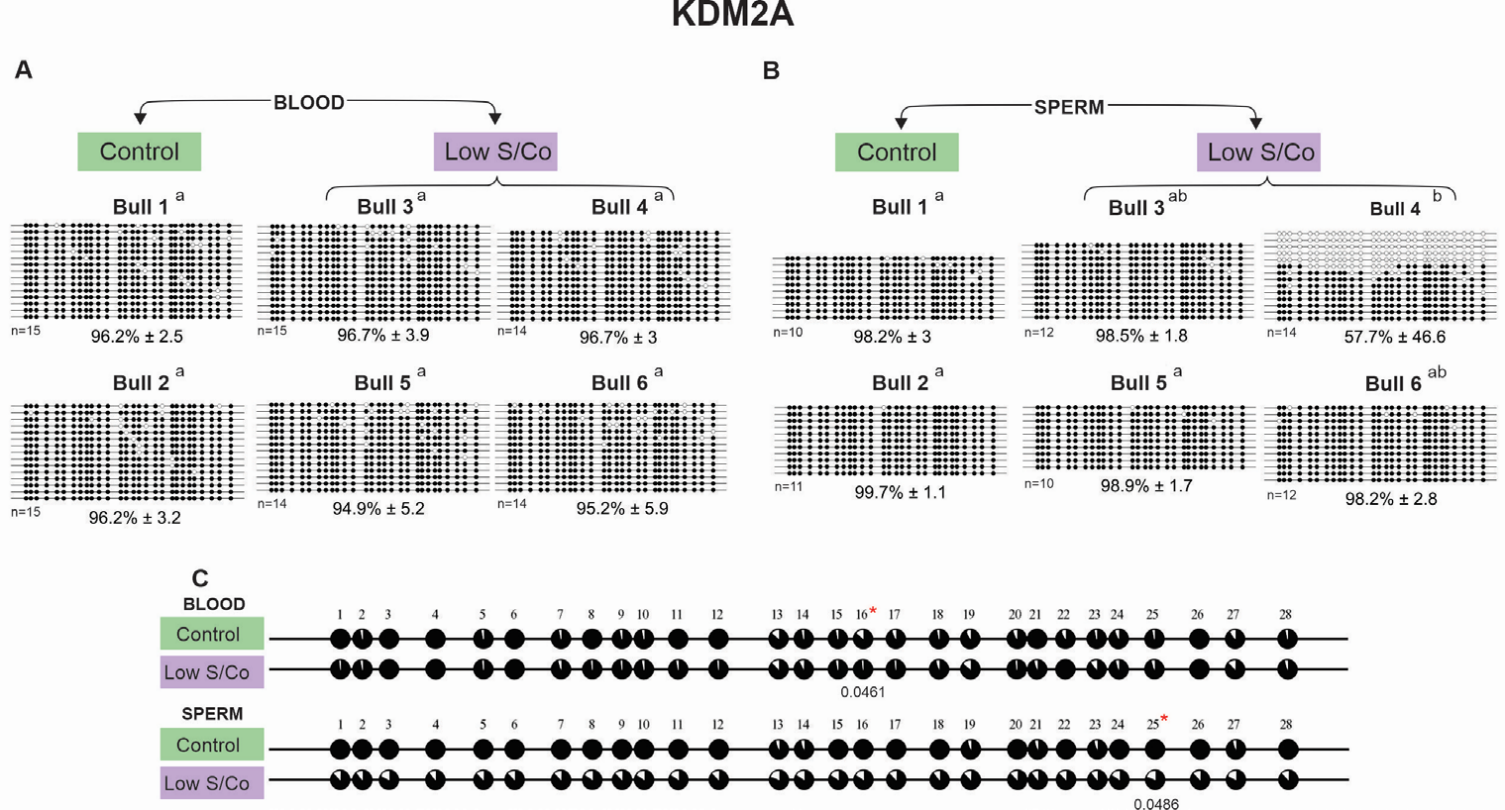
DNA methylation profile of *KDM2A* gene in blood and sperm for control and low S/Co groups. (A) Blood samples, (B) Sperm samples, and (C) Comparative analysis of methylation by CpG sites between control and low S/Co in blood and sperm. Each line represents an individual DNA clone, and each circle represents a CpG dinucleotide. Black circles represent methylated cytosines and white circles represent unmethylated cytosines. The DNA methylation percentage for each animal (Bull 1, Bull 2, Bull 3, Bull 4, Bull 5, and Bull 6) is represented as mean ± standard deviation of the mean. Differences in DNA methylation among animals within the same group are shown by letters a and b (p < 0.05). (*) represents significant difference in the mean values for methylation of individual CpGs using Fisher's exact test (p<0.05). (n) represents the number of sequenced alleles of each sample.

**Figure 3:**
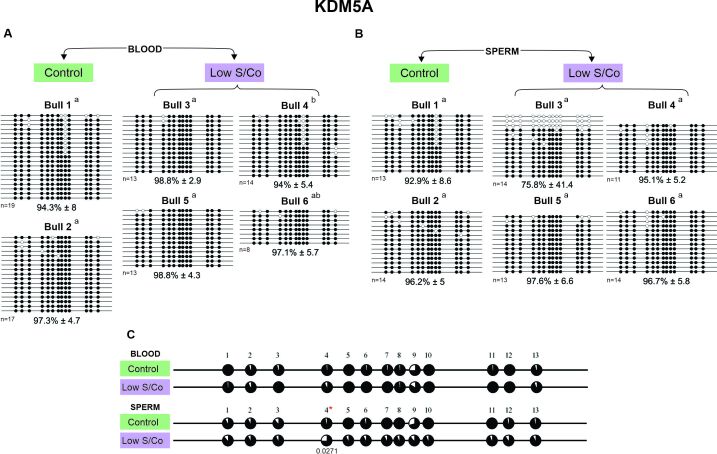
DNA methylation profile of *KDM5A* gene in blood and sperm for control and low S/Co groups. (A) Blood samples, (B) Sperm samples, and (C) Comparative analysis of methylation by CpG sites between control and low S/Co in blood and sperm. Each line represents an individual DNA clone, and each circle represents a CpG dinucleotide. Black circles represent methylated cytosines and white circles represent unmethylated cytosines. The DNA methylation percentage for each animal (Bull 1, Bull 2, Bull 3, Bull 4, Bull 5, and Bull 6) is represented as mean ± standard deviation of the mean. Differences in DNA methylation among animals within the same group are shown by letters a and b (p < 0.05). (*) represents significant difference in the mean values for methylation of individual CpGs using Fisher's exact test (p<0.05). (n) represents the number of sequenced alleles of each sample.

**Figure 4:**
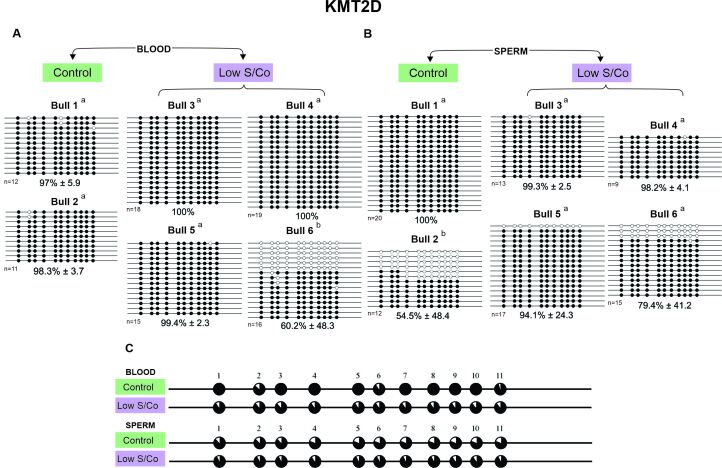
DNA methylation profile of *KMT2D* gene in blood and sperm for control and low S/Co groups. (A) Blood samples, (B) Sperm samples, and (C) Comparative analysis of methylation by CpG sites between control and low S/Co in blood and sperm. Each line represents an individual DNA clone, and each circle represents a CpG dinucleotide. Black circles represent methylated cytosines and white circles represent unmethylated cytosines. The DNA methylation percentage for each animal (Bull 1, Bull 2, Bull 3, Bull 4, Bull 5, and Bull 6) is represented as mean ± standard deviation of the mean. Differences in DNA methylation among animals within the same group are shown by letters a and b (p < 0.05). (n) represents the number of sequenced alleles of each sample.

**Figure 5:**
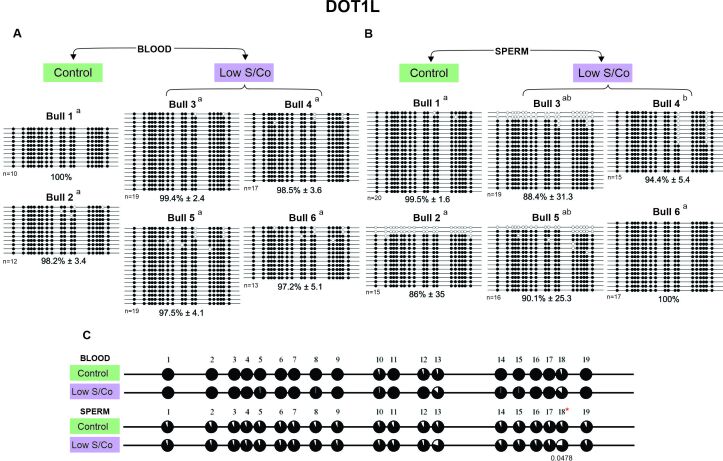
DNA methylation profile of *DOT1L* gene in blood and sperm for control and low S/Co groups. (A) Blood samples, (B) Sperm samples, and (C) Comparative analysis of methylation by CpG sites between control and low S/Co in blood and sperm. Each line represents an individual DNA clone, and each circle represents a CpG dinucleotide. Black circles represent methylated cytosines and white circles represent unmethylated cytosines. The DNA methylation percentage for each animal (Bull 1, Bull 2, Bull 3, Bull 4, Bull 5, and Bull 6) is represented as mean ± standard deviation of the mean. Differences in DNA methylation among animals within the same group are shown by letters a and b (p < 0.05). (*) represents significant difference in the mean values for methylation of individual CpGs using Fisher's exact test (p<0.05). (n) represents the number of sequenced alleles of each sample.

Overall, we analyzed 688 clones and compared the DNA methylation patterns of the four genes *KDM2A*, *KDM5A*, *KMT2D*, and *DOT1L* (detected in the blood and sperm of six Nellore bulls) in the control group against that of the low S/Co groups. The DNA methylation levels (Figures2-5) were classified as low (0-20%), moderate (21-50%), and high (51-100%) according to Zhang et al. (2016) and Silveira et al. (2018).

In general, a hypermethylated pattern was observed in DNA isolated from both the blood and sperm for all genes, groups, and animals (Figures 2-5). However, the *KDM2A* and *KMT2D* genes of three animals showed a lower methylated pattern [*KDM2A*/sperm/low S/Co/Bull 4 (57.7%), Figure 2B; *KMT2D*/blood/low S/Co/Bull 6 (60.2%), Figure 4A; and *KMT2D*/sperm/control/Bull 2 (54.5%), Figure 4B] as the same animal showed alleles with 100% and 0% methylation. We also found more variation in the methylation profile among the sperm alleles in other animals [*KDM5A*/sperm/low S/Co/Bull 3 (75.8%), Figure 3B; *KMT2D*/sperm/low S/Co/Bull 5 (94.1%) and Bull 6 (79.4%), Figure 4B; *DOT1L*/sperm/control/Bull 2 (86%), Figure 5B; *DOT1L*/sperm/low S/Co/Bull 3 (88.4%) and Bull 5 (90.1%), Figure 5B], but it did not influence the higher methylation pattern.

**Figure 6:**
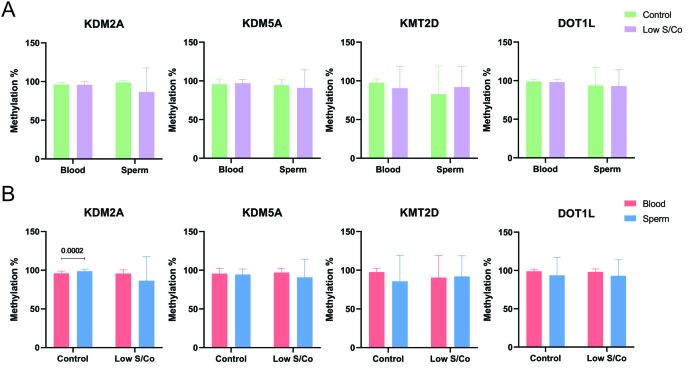
Percentage of methylation in *KDM2A*, *KDM5A*, *KMT2D*, and *DOT1L* genes (A) Comparison of DNA methylation levels between control and low S/Co groups for blood and sperm samples. (B) Comparison of DNA methylation levels in blood and sperm samples in the control and low S/Co groups, respectively. Numbers represent significant differences in the mean values for methylation using the Mann-Whitney test (p ≤ 0.05)

Interestingly, when we compared the methylation status of each CpG site individually, we found specific CpGs differentially methylated between control and low S/Co for *KDM2A* in the blood (CpG 17) and sperm (CpG 25) (Figure 2C), for *KDM5A* in sperm (CpG 4) (Figure 3C), and *DOT1L* in sperm (Figure 5C). However, when we compared all CpG sites among themselves, there were no differences in DNA methylation patterns for any of the genes between the control and low S/Co groups in the blood or sperm samples (Figure 6A). Therefore, treatment with a low S/Co diet in the heifers during the pre-and periconceptional periods did not affect the DNA methylation pattern of the gamete and blood cells of the progeny in adulthood for the DMRs analyzed.

We also analyzed DNA methylation in the blood and sperm tissues of the control and low S/Co groups and found a difference in DNA methylation only in the control groups for KDM2A (Figure 6B); however, despite the statistical difference between the tissues analyzed, all samples were considered hypermethylated.

## Discussion

In mammals, extensive epigenomic remodelling occurs during the initial stages of development. During gametogenesis and embryogenesis, epigenetic marks are more vulnerable to environmental influences. In our previous study, we presented DMR candidates for investigation, focusing on the impact of maternal nutrition on foetal epigenetic reprogramming during the pre- and peri-conceptional periods (Nochi et al., 2022). Therefore, to validate whether DMRs in blastocysts are maintained in adulthood, we characterized four DMRs in the sperm and blood from F1 animals.

Our previous study applied the experimental diet during the *de novo* methylation phase of F0 gametogenesis after the animals reached puberty (Nochi et al., 2022). Although Nochi et al. (2022) found an altered DNA methylation pattern in the blastocyst stage between the low S/Co and control groups in their study, we identified a hypermethylated pattern for both groups in all the DMRs analyzed in both the blood and sperm DNA of F1. This result suggests that embryos from both groups reprogrammed their epigenetic profiles correctly in the blood and sperm cells during development. Thus, epigenetic reprogramming during embryogenesis prevents the transmission of F0 gametic epimutations to F1.

Despite a second wave of epigenetic reprogramming, some regions are not reprogrammed during embryogenesis. The DMRs established during gametogenesis are known as germ line DMRs (gDMRs). Those that are reprogrammed are known as transient DMRs (tDMRs) (Proudhon et al., 2012; Smallwood and Kelsey, 2012). In contrast, the imprinted DMRs (iDMRs) are those DMRs that are protected from loss of methylation after fertilization and are not methylated during embryo or tissue differentiation (Proudhon et al., 2012; MacDonald and Mann, 2014; Thakur et al., 2016). In our study model, the diet on final gametogenesis did not exert a permanent effect in the DMRs studied. However, since iDMRs are protected from reprogramming during embryogenesis, if the diet had affected these DMRs in any way, those effects probably would have been retained into adulthood to create metastable epialleles.

In addition to evaluating DNA methylation patterns in white blood cells in F1 adults, we also analysed DNA methylation in the sperm cells of these animals. Despite the hypermethylated state in the blood and sperm samples, we found more variation in DNA methylation patterns among sperm alleles. Interestingly, several studies in humans and cattle have described the potential use of sperm DNA methylation-epimutations as biomarkers of infertility and susceptibility to diseases (Kropp et al., 2017; Nasri et al., 2017; Capra et al., 2019; Lujan et al., 2019; Garrido et al., 2021). Thus, further studies characterizing whether maternal diet can influence the sperm DNA methylation of the offspring will provide valuable information.

A previous study in humans revealed some sensitive environmental hotspots in the embryonic methylome (Silver et al., 2022). In contrast, a low S/Co diet administered during gametogenesis stochastically affected the embryonic methylome (Nochi et al., 2022). Thus, the regions of the embryonic epigenome that are impacted by the dietary effects may be reprogrammed without deleterious changes in the offspring.

It is well known that the diet during pregnancy may affect the offspring. Several studies have confirmed the effect of different maternal diets on the offspring in humans (Roseboom et al., 2006), mice (Guo et al., 2018; Mao et al., 2018; Xie et al., 2018), rats (Carlin et al., 2019; Pedrana et al., 2020), and bovines (Devos et al., 2021; Liu et al., 2021; Noya et al., 2021). Moreover, studies reported that the maternal diet during gestation affects the DNA methylation pattern in the placenta and offspring of mice (Ge et al., 2014; Mahajan et al., 2019; Zhang et al., 2021a), cattle (Liu et al., 2021), and humans (Daniels et al., 2020; Kupers et al., 2022). Interestingly, a study showed that the exposure of IVP embryos to choline in the culture medium alters the DNA methylation profile in the offspring muscle (Estrada-Cortes et al., 2021). However, these studies evaluated the effects during embryogenesis. DMRs, by contrast, are more likely to propagate in the tissue of the offspring when the stimuli affect epigenetic reprogramming beyond the stage of gametogenesis but also embryogenesis. Therefore, determining the time and duration to which the dietary stimuli exert its effect is essential to study and understand the consequences of the maternal diet on the offspring.

Interestingly, studies in livestock have only evaluated the dietary effects during the final stages of *de novo* methylation (Sinclair et al., 2007; Zglejc-Waszak et al., 2019; Toschi et al., 2020), but Nochi et al. (2022) evaluated the impact of nutrition starting from the initial stage of *de novo* methylation during gametogenesis. A broad experimental design for the study of environmental influence on gametogenesis should contemplate the erasure of DNA methylation during foetal programming and ensure the dietary effect on oocytes during *de novo* methylation. However, this experimental design is easier to implement in mice models than it is in cattle because of its expensive and time-consuming nature. Moreover, based on our observations, embryonic methylome can be used only as an initial screening tool because DMRs may be reprogrammed in the final stages of *de novo* methylation during embryogenesis and foetal growth; therefore, it is crucial to validate the DMRs in adulthood.

## Conclusion

In this study, we characterized the DMRs identified in the previous experiment, which showed that the pre-and periconceptional diet affected the DNA methylation profile of embryos. Among the 2,320 DMRs identified in blastocysts by Nochi et al. (2022), the six that we analyzed underwent extensive epigenetic reprogramming in both blood and sperm cells. These results confirm our hypothesis that the DMRs found in embryos may not be maintained in adult animals. Thus, we suggest that after the first screening using WGBS, it is crucial to confirm the inheritance before projecting the embryonic methylome onto adult tissues.
